# Supplementary material for: Transcriptome Profiling Provides New Insights into the Molecular Mechanism Underlying the Sensitivity of Cotton Varieties to Mepiquat Chloride
Source: Int J Mol Sci. 2022 May 2;23(9):5043. doi: 10.3390/ijms23095043 (PMC9105546; doi:10.3390/ijms23095043)
Supplement: Supplementary file 1 [file ijms-23-05043-s001.zip › ijms-1588448-supplementary/Figure S1-S3.pdf]

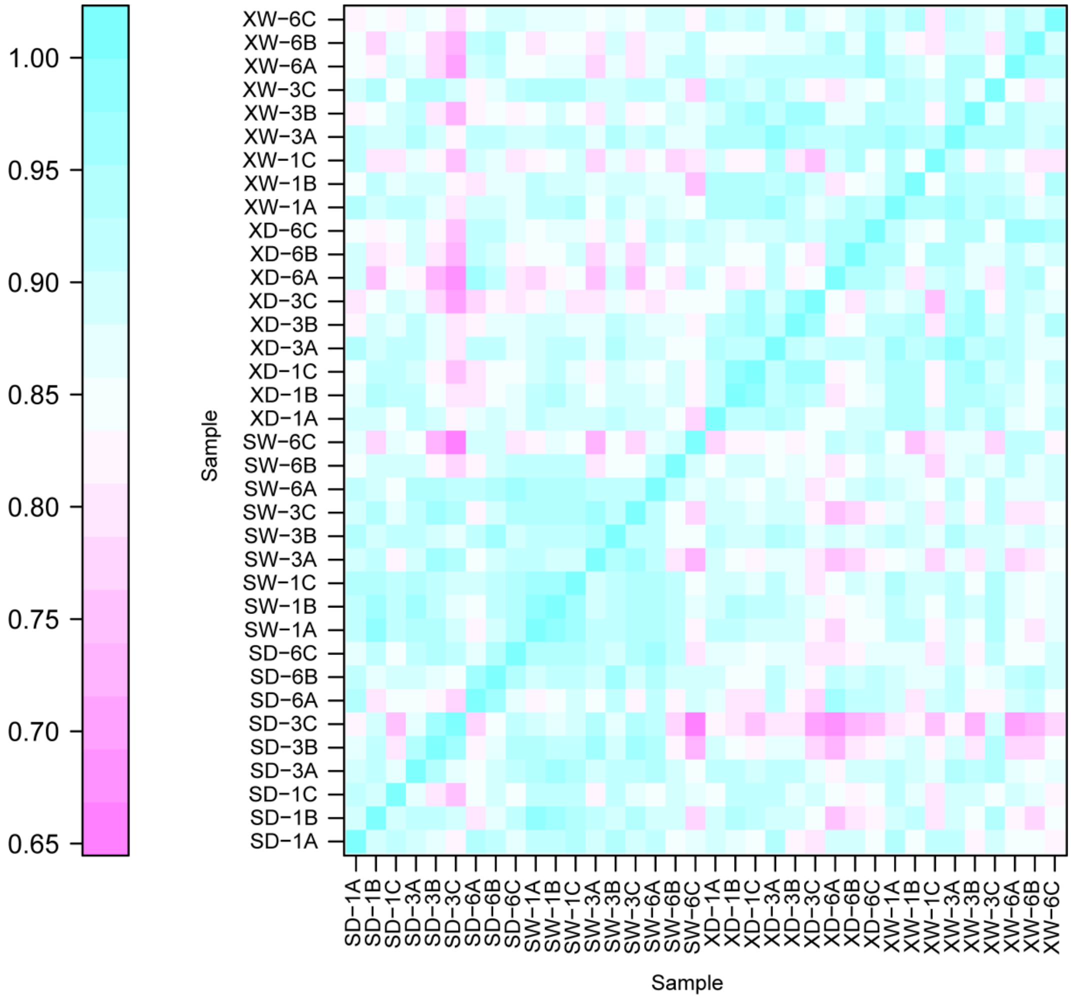

Figure S1.Pearson correlation between 36 samples

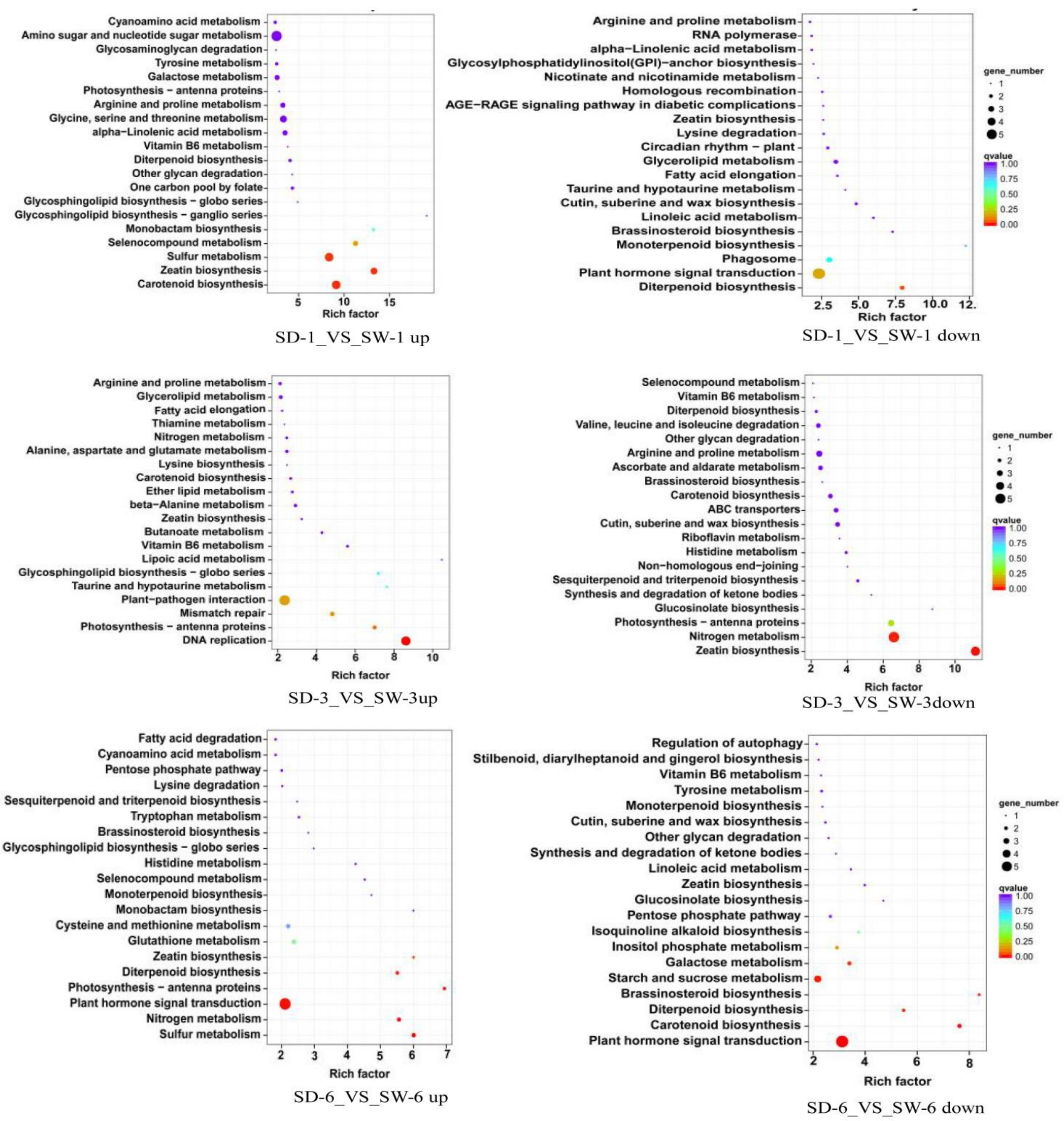

Figure S2. KEGG pathway enrichment analysis of up and down-regulated DEGs between MC and Water treatment at 3 time point in SD1068. Y-axis shows the KEGG pathways, and X-axis shows the Rich factor. The size and color of each circle represent the number of enriched genes in the corresponding pathway and the -log10 (q value) value, respectively. SD-1, SD-3 and SD-6 represent samples at 1, 3 and 6 dps with MC treatment, respectively. SW-1, SW-3 and SW-6 represent samples at 1, 3 and 6 dps with water treatment, respectively.

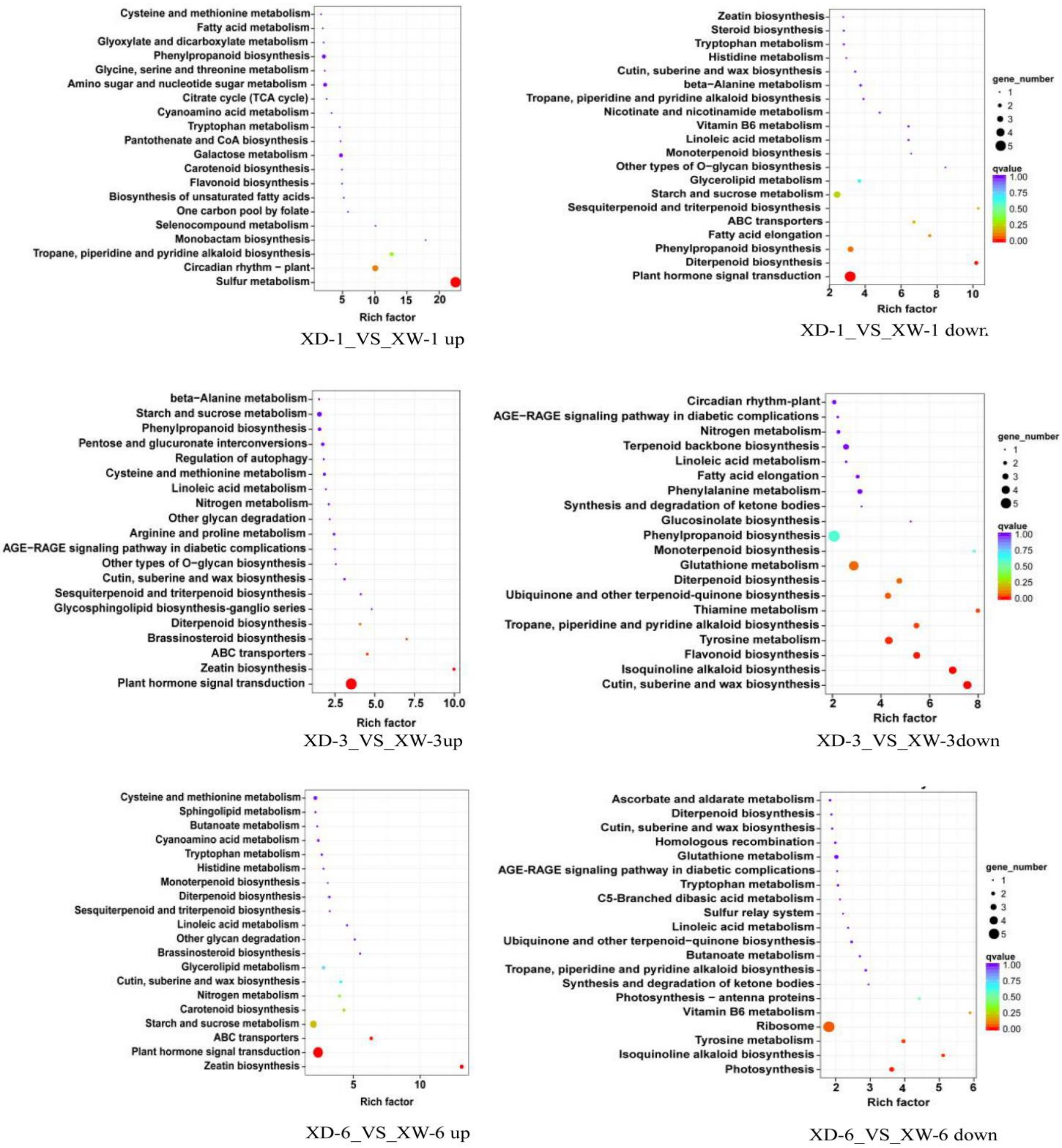

Figure S3. KEGG pathway enrichment analysis of up and down-regulated DEGs between MC and Water treatment at 3 time point in XLZ74. Y-axis shows the KEGG pathways, and X-axis shows the Rich factor. The size and color of each circle represent the number of enriched genes in the corresponding pathway and the -$\log_{10}$ $^{(q\ value)}$ value, respectively. XD-1, XD-3 and XD-6 represent samples at 1, 3 and 6 dps with MC treatment, respectively. XW-1, XW-3 and XW-6 represent samples at 1, 3 and 6 dps with Water treatment, respectively.
